# Supplementary material for: Single cell profiling of primary and paired metastatic lymph node tumors in breast cancer patients
Source: Nat Commun. 2022 Nov 10;13:6823. doi: 10.1038/s41467-022-34581-2 (PMC9649678; doi:10.1038/s41467-022-34581-2)
Supplement: Supplementary file 1 — Supplementary Information [file 41467_2022_34581_MOESM1_ESM.pdf]

Supplementary Information

Single Cell Profiling of Primary and Paired Metastatic Lymph Node Tumors in Breast Cancer Patients

Tong Liu[1#], Cheng Liu[2,3,4#], Meisi Yan[5#], Lei Zhang[2,3,4], Jing Zhang[2,3,4], Min Xiao[1], Zhigao Li[1*], Xiaofan Wei[2,3,4*], Hongquan Zhang[2,3,4,6*]

1. Department of Breast Surgery, Harbin Medical University Cancer Hospital, Harbin, China; Heilongjiang Academy of Medical Sciences, Harbin, China;
2. Program for Cancer and Cell Biology, Department of Human Anatomy, Histology and Embryology, School of Basic Medical Sciences, Peking University Health Science Center, Beijing 100191, China;
3. Peking University International Cancer Institute, Peking University Health Science Center, Beijing 100191, China;
4. MOE Key Laboratory of Carcinogenesis and Translational Research and State Key Laboratory of Natural and Biomimetic Drugs, Peking University Health Science Center, Beijing 100191, China;
5. Department of Pathology, Harbin Medical University, Harbin 150081, China;
6. Department of Human Anatomy, Histology, and Embryology, Shenzhen University School of Medicine, Shenzhen 518055, China.

[#]These authors contributed equally to this paper.

*Corresponding authors: Hongquan Zhang, E-mail: Hongquan.Zhang@bjmu.edu.cn; Xiaofan Wei, E-mail: weixiaofan@bjmu.edu.cn; Zhigao Li, E-mail: drzhigaoli@hrbmu.edu.cn.

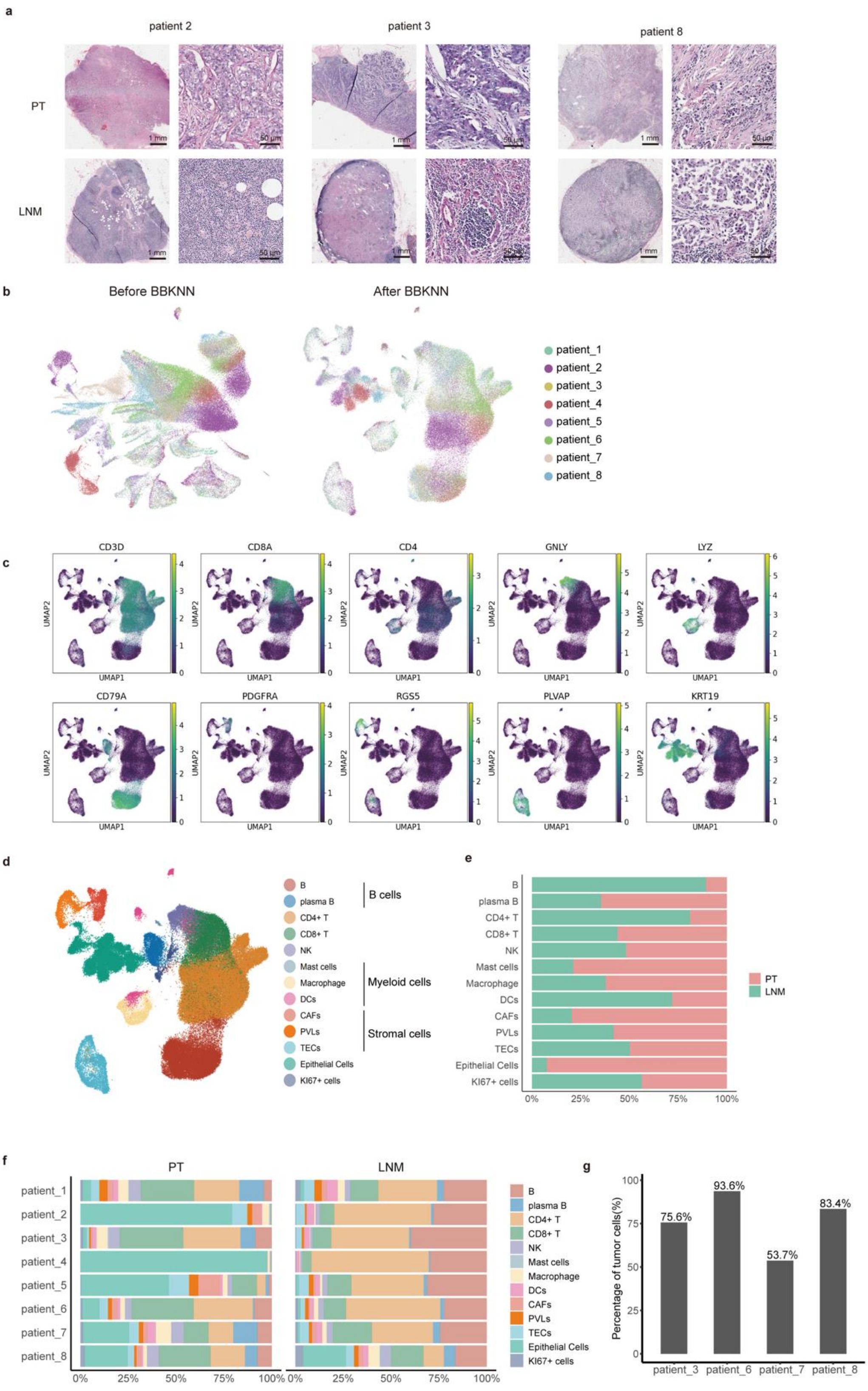

a
patient 2
patient 3
patient 8
PT
LNM
1 mm
50 μm
b
Before BBKNN
After BBKNN
patient_1
patient_2
patient_3
patient_4
patient_5
patient_6
patient_7
patient_8
c
CD3D
CD8A
CD4
GNLY
LYZ
CD79A
PDGFRA
RGS5
PLVAP
KRT19
UMAP1
UMAP2
d
B
plasma B
CD4+ T
CD8+ T
NK
Mast cells
Macrophage
DCs
CAFs
PVLs
TECs
Epithelial Cells
KI67+ cells
B cells
Myeloid cells
Stromal cells
e
PT
LNM
0%
25%
50%
75%
100%
f
PT
LNM
patient_1
patient_2
patient_3
patient_4
patient_5
patient_6
patient_7
patient_8
g
Percentage of tumor cells(%)
75.6%
93.6%
53.7%
83.4%
patient_3
patient_6
patient_7
patient_8

Supplementary Fig. 1. Overview of major clusters in PT and MLNT (related to Fig. 1)

a. Hematoxylin and eosin (HE) staining of PT and MLNT tissues in 3 representative patients. Original magnification, 10x , scale bar = 1 mm or 200x , scale bar = 50 μm**. b**. UMAP visualization of the entire data set before (left) and after (right) batch alignment. Cells were colored by batch effect (patients). **c&d**. UMAP plots showing the expression pattern of marker genes (c) or major cell types (d). **e**. The proportion of tissues within major cell types. The color represents different tissues. The *x-axis* is the fraction of tissues, and the *y-axis* is the major cell types. **f**. The proportion of major cell types in tumor samples (left) or lymph node metastasis (right). The color represents the cell types. The *x-axis* is the fraction of cell types, and the *y-axis* presents different patients. **g.** Percentage of spots representing positive detected tumor cells in LNMT. Source data are provided as a Source Data file.

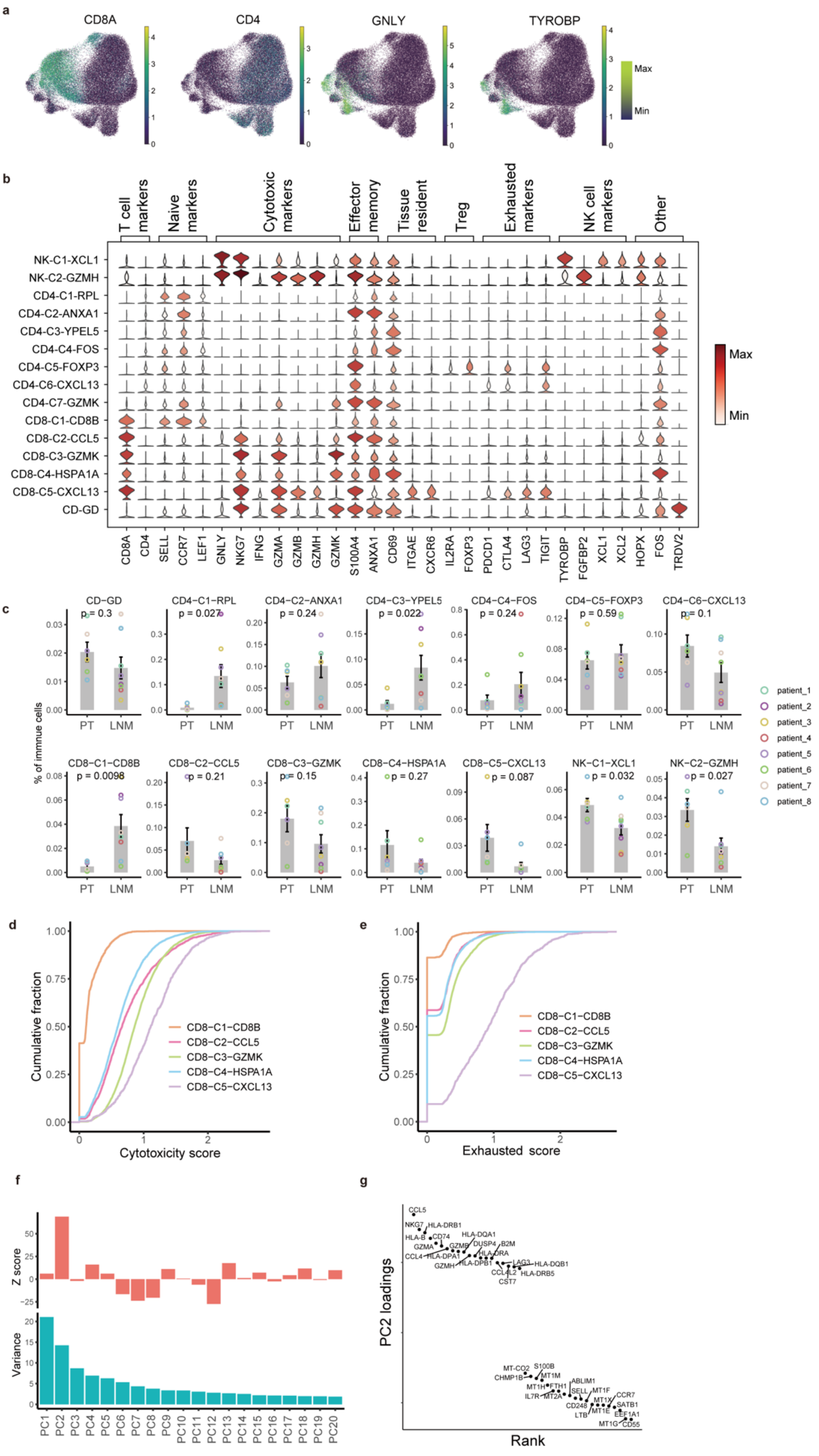

a
CD8A
CD4
GNLY
TYROBP
Max
Min
b
T cell markers
Naive markers
Cytotoxic markers
Effector memory
Tissue resident
Treg
Exhausted markers
NK cell markers
Other
NK-C1-XCL1
NK-C2-GZMH
CD4-C1-RPL
CD4-C2-ANXA1
CD4-C3-YPEL5
CD4-C4-FOS
CD4-C5-FOXP3
CD4-C6-CXCL13
CD4-C7-GZMK
CD8-C1-CD8B
CD8-C2-CCL5
CD8-C3-GZMK
CD8-C4-HSPA1A
CD8-C5-CXCL13
CD-GD
CD8A CD4 SELL CCR7 LEF1 GNLY NKG7 IFNG GZMA GZMB GZMH GZMK S100A4 ANXA1 CD69 ITGAE CXCR6 IL2RA FOXP3 PDCD1 CTLA4 LAG3 TIGIT TYROBP FGFBP2 XCL1 XCL2 HOPX FOS TRDV2
Max
Min
c
% of immnue cells
CD−GD p = 0.3
CD4−C1−RPL p = 0.027
CD4−C2−ANXA1 p = 0.24
CD4−C3−YPEL5 p = 0.022
CD4−C4−FOS p = 0.24
CD4−C5−FOXP3 p = 0.59
CD4−C6−CXCL13 p = 0.1
CD8−C1−CD8B p = 0.0098
CD8−C2−CCL5 p = 0.21
CD8−C3−GZMK p = 0.15
CD8−C4−HSPA1A p = 0.27
CD8−C5−CXCL13 p = 0.087
NK−C1−XCL1 p = 0.032
NK−C2−GZMH p = 0.027
PT LNM
patient_1
patient_2
patient_3
patient_4
patient_5
patient_6
patient_7
patient_8
d
Cumulative fraction
Cytotoxicity score
CD8−C1−CD8B
CD8−C2−CCL5
CD8−C3−GZMK
CD8−C4−HSPA1A
CD8−C5−CXCL13
e
Cumulative fraction
Exhausted score
CD8−C1−CD8B
CD8−C2−CCL5
CD8−C3−GZMK
CD8−C4−HSPA1A
CD8−C5−CXCL13
f
Z score
Variance
PC1 PC2 PC3 PC4 PC5 PC6 PC7 PC8 PC9 PC10 PC11 PC12 PC13 PC14 PC15 PC16 PC17 PC18 PC19 PC20
g
PC2 loadings
Rank
CCL5
NKG7
HLA-DRB1
HLA-B
CD74
HLA-DQA1
GZMA
GZMB
DUSP4
B2M
CCL4
HLA-DPA1
HLA-DRA
GZMH
HLA-DPB1
LAG3
HLA-DQB1
CCL4L2
HLA-DRB5
CST7
MT-CO2
S100B
CHMP1B
MT1M
MT1H
FTH1
ABLIM1
SELL
MT1F
IL7R
MT2A
CCR7
CD248
MT1X
SATB1
LTB
MT1E
EEF1A1
MT1G
CD55

Supplementary Fig. 2. Characterization of T cells and NK cells in PT and MLNT (related to Fig. 2)

a. A UMAP embedding plot showing representative markers of T cells and NK cells. **b.** Violin plots show a set of known marker genes that distinguish various clusters. **c**. Bar plots showing the percentages of T cells and NK cell clusters between 2 tissues (PT: n = 8 samples, LNM: n = 8 samples). Statistical testing was performed by two-sided Wilcoxon test. Data are presented as mean values +/- SD. **d&e**. Cumulative distribution plot showing the distribution of cytotoxic scores (e) or exhausted scores (f) in each CD8 T subtype. A rightward shift of the curve indicates increased state scores. **f.** The variance (bottom) and *z* score (top) of the first 20 PCs. The expression matrix of CD8 T cells from PT and MLNT was calculated for the first 20 PCs. To compare the difference between PTs and MLNTs in each PC, *z* scores from unpaired *t* test were measured between the tumor and lymph node of each PC. **g**. The top 20 genes that contribute to PC2 variance. Genes were ranked by PC2 loadings. Source data are provided as a Source Data file.

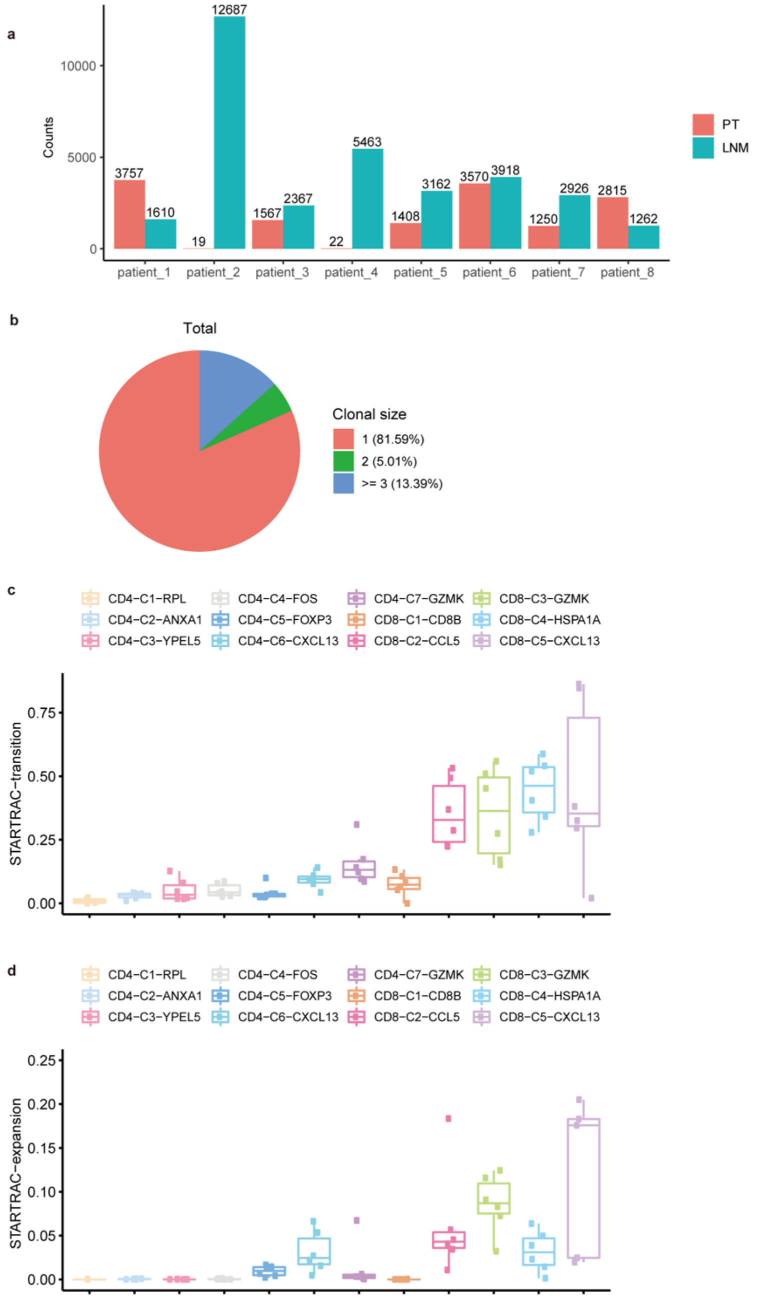

Supplementary Fig. 3. Characterization of TCR in PT and MLNT (related to Fig. 3)

a. Bar plot showing the number of T cells for TCR analysis in each patient. Very rare immune cells were present in Patient 2 and Patient 4; thus, these 2 samples were removed from downstream analysis. **b.** Pie charts showing the fraction of clonal size of all clonotypes. Red represents unique clonotypes, green represents clonal size clonotypes of 2, and blue represents clonal size clonotypes of 3 or above. **c&d**. Developmental transition ability (b) or clonal expansion levels (c) of CD4 T cells and CD8 T cells clusters quantified by STARTRAC-transition indices for each patient (n = 6). In the box plots, the center line corresponds to the median, box corresponds to the interquartile range (IQR), and whiskers 1.5 × IQR. Source data are provided as a Source Data file.

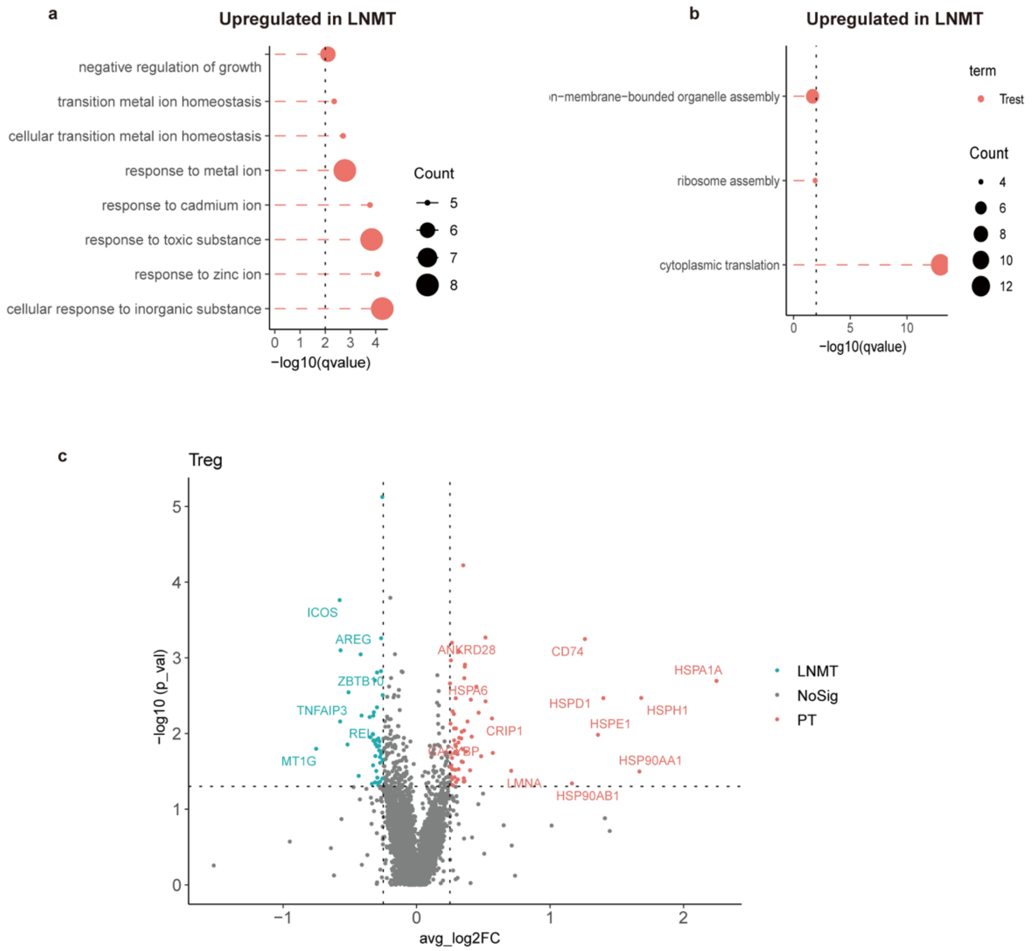

Supplementary Fig. 4. Characterization of migrated T cells in PT and MLNT (related to Fig. 4)

a&b. Top 8 enriched pathways for up-regulated genes for matched CD8 T cells (a) or matched Tconvs (b) in LNMT vs PT. **c**. Volcano plot showing differentially expressed genes between LNMT vs PT for matched Tregs. *P* value <0.05, log 2 (fold change) ≥ 0.25. Statistical testing was performed by a two-sided Wilcoxon test. Source data are provided as a Source Data file.

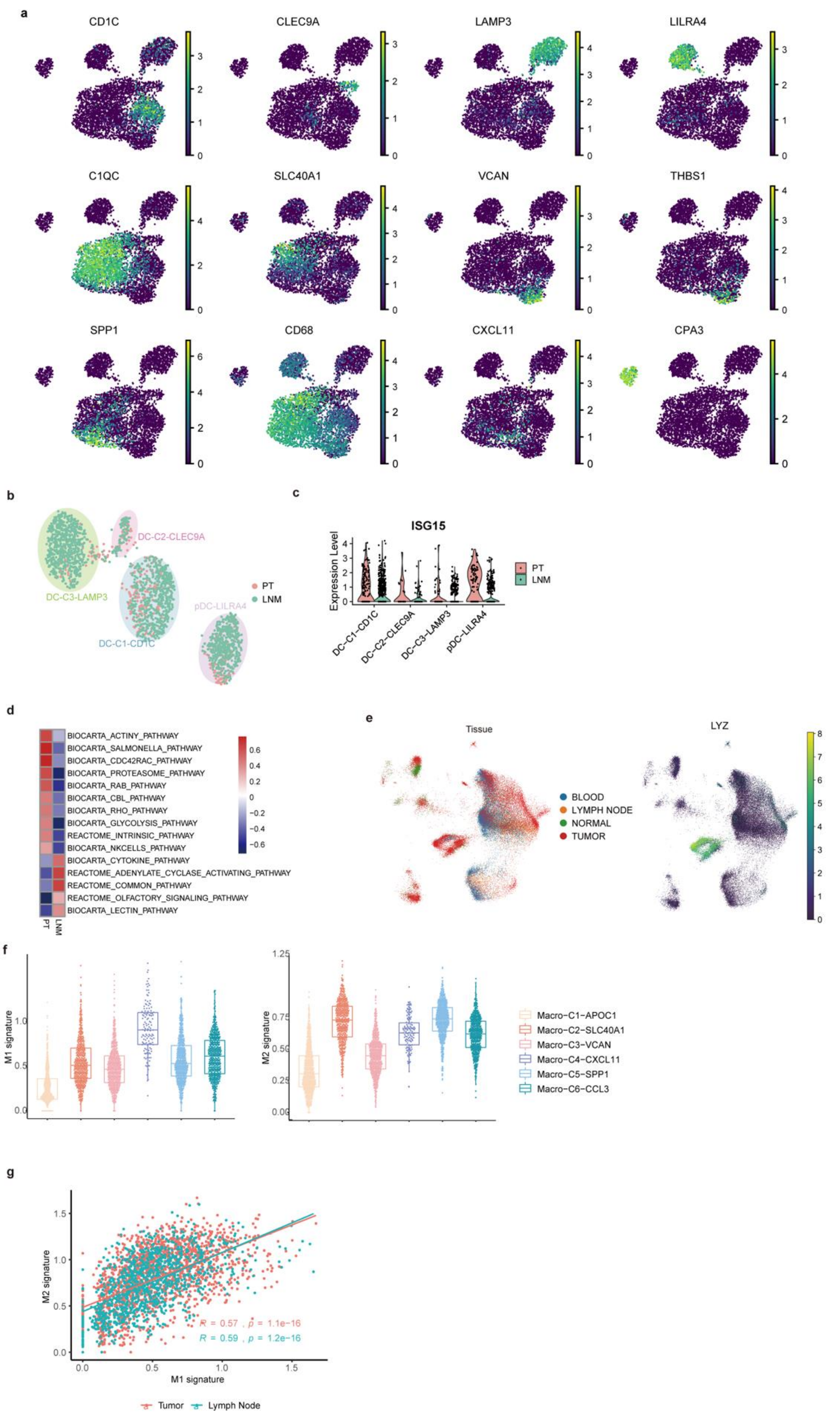
a
CD1C
CLEC9A
LAMP3
LILRA4
C1QC
SLC40A1
VCAN
THBS1
SPP1
CD68
CXCL11
CPA3
b
DC-C2-CLEC9A
DC-C3-LAMP3
DC-C1-CD1C
pDC-LILRA4
PT
LNM
c
ISG15
Expression Level
DC-C1-CD1C
DC-C2-CLEC9A
DC-C3-LAMP3
pDC-LILRA4
PT
LNM
d
BIOCARTA_ACTINY_PATHWAY
BIOCARTA_SALMONELLA_PATHWAY
BIOCARTA_CDC42RAC_PATHWAY
BIOCARTA_PROTEASOME_PATHWAY
BIOCARTA_RAB_PATHWAY
BIOCARTA_CBL_PATHWAY
BIOCARTA_RHO_PATHWAY
BIOCARTA_GLYCOLYSIS_PATHWAY
REACTOME_INTRINSIC_PATHWAY
BIOCARTA_NKCELLS_PATHWAY
BIOCARTA_CYTOKINE_PATHWAY
REACTOME_ADENYLATE_CYCLASE_ACTIVATING_PATHWAY
REACTOME_COMMON_PATHWAY
REACTOME_OLFACTORY_SIGNALING_PATHWAY
BIOCARTA_LECTIN_PATHWAY
PT
LNM
e
Tissue
LYZ
BLOOD
LYMPH NODE
NORMAL
TUMOR
f
M1 signature
M2 signature
Macro−C1−APOC1
Macro−C2−SLC40A1
Macro−C3−VCAN
Macro−C4−CXCL11
Macro−C5−SPP1
Macro−C6−CCL3
g
M2 signature
M1 signature
R = 0.57 , p = 1.1e−16
R = 0.59 , p = 1.2e−16
Tumor
Lymph Node

Supplementary Fig. 5. Myeloid cells in PT and MLNT (related to Fig. 5).

a. A UMAP plot showing the expression pattern of selected marker genes for myeloid clusters. **b.** A UMAP plot showing DC clusters color-coded by tissues. Different cell clusters are circled. **c**. A violin plot showing the *ISG15* expression level of DC clusters. **d**. A heatmap showing the difference of enriched pathway analyzed by GSVA between PT and MLNT in the DC-C3-*LAMP3* cluster. **e**. A UMAP plot showing the projection of GSE114727 dataset[1] according to the "ingest" function. Cells are colored according to tissue type (left) or myeloid markers, *LYZ* (right). **f**. A boxplot of the M1 (left panel) and M2 (right panel) signature of macrophage clusters. (Macro-C1-*APOC1* n = 627 cells, Macro-C2-*SLC40A1* n = 468 cells, Macro-C3-*VCAN* n = 540 cells, Macro-C4-*CXCL11* n = 126 cells, Macro-C5-*SPP1* n = 542 cells, Macro-C6-*CCL3* = 475 cells). In the box plots, the center line corresponds to the median, the box corresponds to the IQR, and whiskers 1.5 × IQR. **g**. Scatter plots show that M1 correlated with the M2 signature in PTs and LNMTs. The correlation coefficient and *P*-value were calculated with two-sided Pearson rank correlation. Source data are provided as a Source Data file.

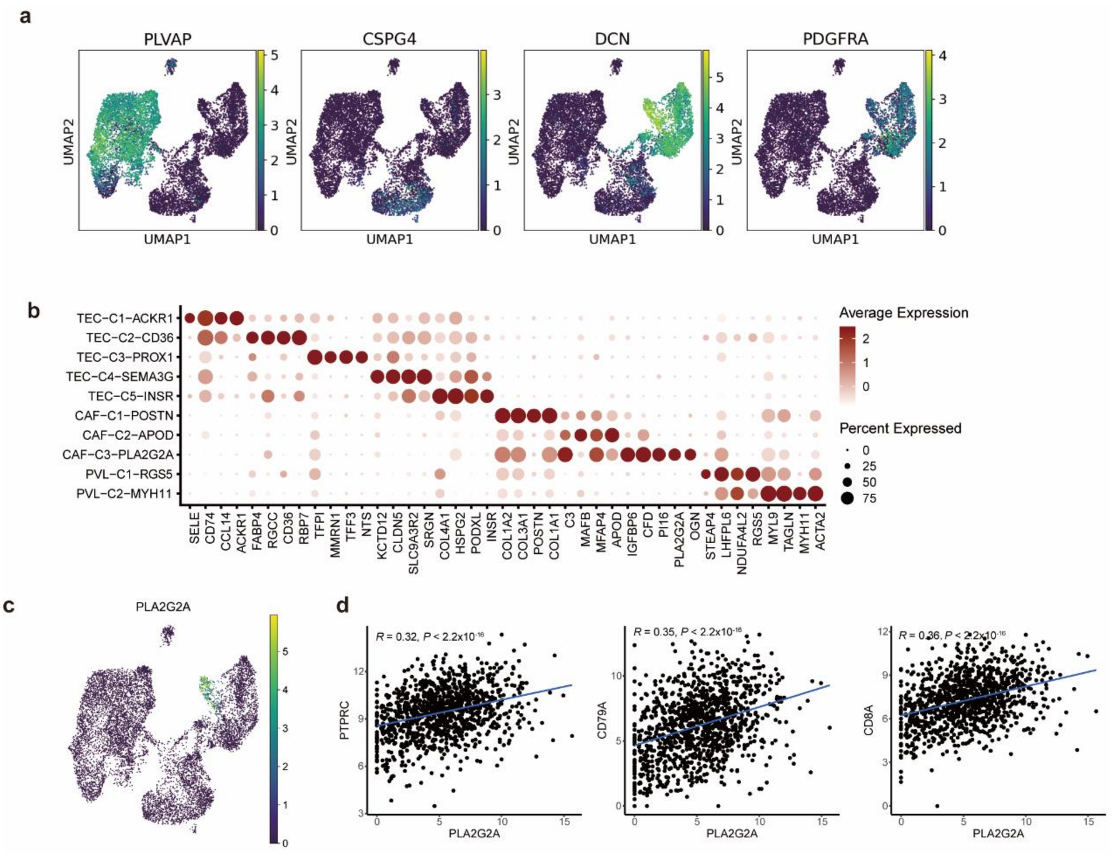

Supplementary Fig. 6. Characterization of PLA2G2A+ CAFs (related to Fig. 6)

a. A UMAP plot showing the marker genes for 3 types of stromal cells. Yellow indicates higher expression and black indicates lower expression. **b**. A dot plot showing marker genes across TEC and CAF clusters. Dot sizes indicate the fraction of expressed cells and are colored according to the normalized expression level. **c**. A UMAP plot showing the *PLA2G2A* in the TEC and CAF data sets. Yellow indicates higher expression and black indicates lower expression. **d**. Scatter plots showing *PLA2G2A* correlated with immune cell markers in the TCGA-BRCA dataset (n = 640). Lines are fit for each select pair. The correlation coefficient and *P*-value were calculated with two-sided Pearson rank correlation. Source data are provided as a Source Data file.

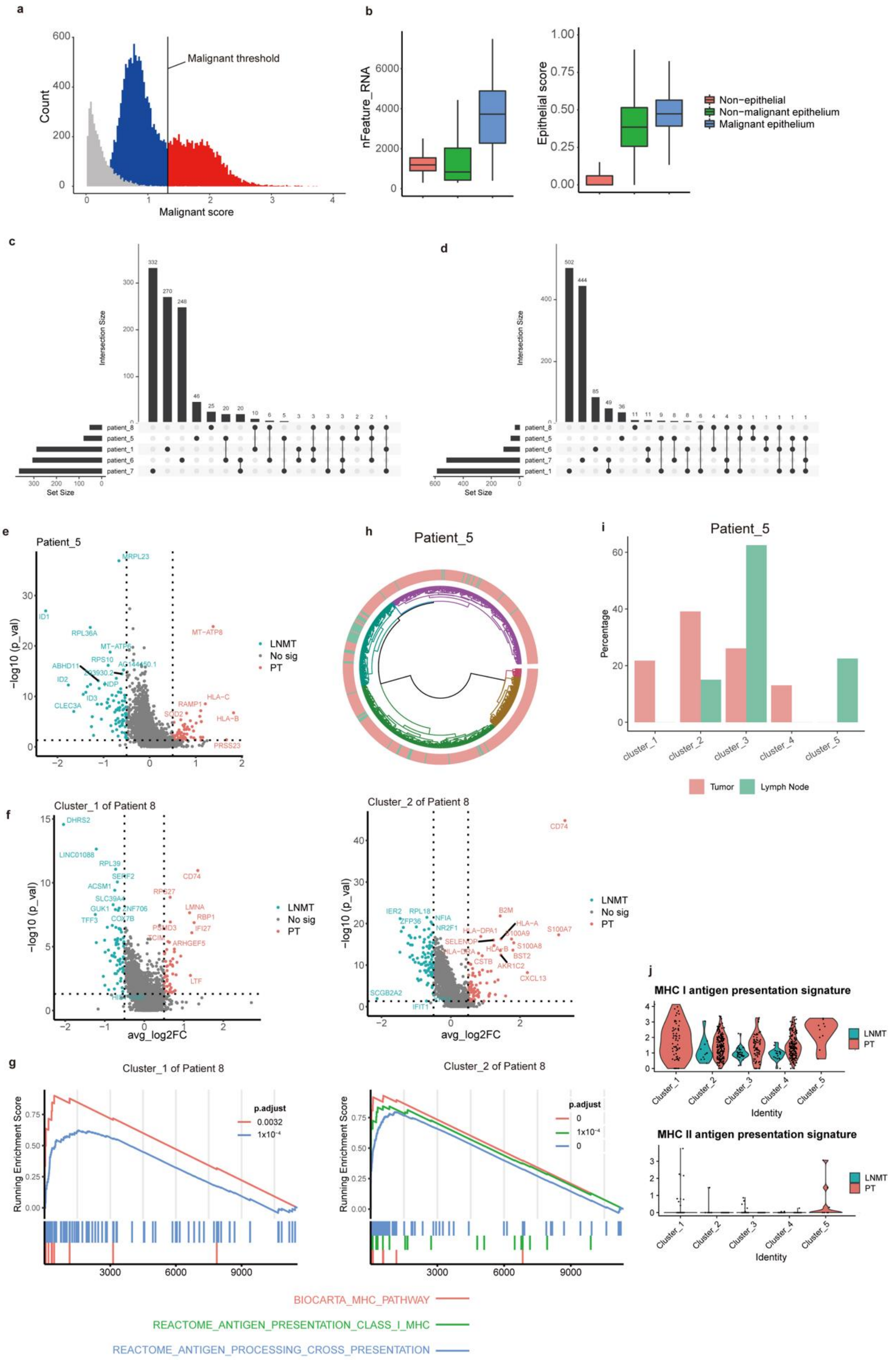
a
Malignant threshold
Count
Malignant score
b
nFeature_RNA
Epithelial score
Non−epithelial
Non−malignant epithelium
Malignant epithelium
c
Intersection Size
Set Size
d
e
Patient_5
LNMT
No sig
PT
h
Patient_5
i
Patient_5
Percentage
Tumor
Lymph Node
f
Cluster_1 of Patient 8
Cluster_2 of Patient 8
avg_log2FC
−log10 (p_val)
j
MHC I antigen presentation signature
MHC II antigen presentation signature
Identity
g
Cluster_1 of Patient 8
Cluster_2 of Patient 8
Running Enrichment Score
p.adjust
BIOCARTA_MHC_PATHWAY
REACTOME_ANTIGEN_PRESENTATION_CLASS_I_MHC
REACTOME_ANTIGEN_PROCESSING_CROSS_PRESENTATION

Supplementary Fig. 7. Characterization of epithelial cells (related to Fig. 7)

a. A histogram showing the distribution of malignant scores in epithelial cells and reference cells. Orange indicates the malignant score distribution of malignant epithelial cells, blue indicates nonmalignant epithelial cells, and grey indicates reference cells. **b**. Cells were classified into malignant epithelial cells, nonmalignant cells, and non-epithelial cells. A boxplot the showing number of features (left) and epithelial scores (right) of the 3 types. (Non-epithelial: n= 100422 cells, Non-malignant epithelium: n = 12069 cells, Malignant epithelium: n = 6354 cells). In the box plots, the center line corresponds to the median, the box corresponds to the IQR, and whiskers 1.5 × IQR. **c&d**. Upset plot showing the overlapping of the upregulated genes in PT (c) and the upregulated genes in LNMT (d). Differential analysis was calculated for each patient. *P* value < 0.05, log2 (fold change) ≥ 0.5. **e**. Volcano plot showing the differentially expressed genes between PT and LNMT in malignant cells from patient 5. *P* value < 0.05, log2 (fold change) ≥ 0.5. Statistical testing was performed by a two-sided Wilcoxon test. **f&g**. Volcano plot showing the differentially expressed genes between PT and LNMT in malignant cells for CNV cluster 1(f) or CNV cluster 2 (g) from patient 8. *P* value < 0.05, log2 (fold change) ≥ 0.5. Statistical testing was performed by a two-sided Wilcoxon test. g. GSEA analysis showed that genes rank in PT vs LNMT of cluster 1 (left panel) and cluster 2 (right panel) in patient 8 are enriched in the antigen presentation pathway. The left represents a high gene rank in PT. Statistical testing was performed by permutation test. The *P*-values were corrected with Benjamini-Hochberg adjustment. **h**. Circos plot showing 6 CNV clusters of malignant epithelial cells according to CNVs similarity for patient 5. Cells were colored by tissue. i. The bar plot shows the frequency of 5 CNV clusters in patient 5. **j**. Violin plots showing the MHC I antigen presentation signature (Up) and MHC II antigen presentation signature (Down) of 5 CNV clusters from patient 5. Source data are provided as a Source Data file.

Supplementary References:

1 Azizi, E. *et al.* Single-Cell Map of Diverse Immune Phenotypes in the Breast Tumor Microenvironment. *Cell* **174**, 1293-1308 e1236, doi:10.1016/j.cell.2018.05.060 (2018).
