# Supplementary material for: Single cell profiling of primary and paired metastatic lymph node tumors in breast cancer patients
Source: Nat Commun. 2022 Nov 10;13:6823. doi: 10.1038/s41467-022-34581-2 (PMC9649678; doi:10.1038/s41467-022-34581-2)
Supplement: Supplementary file 3 — Description of Additional Supplementary Files [file 41467_2022_34581_MOESM3_ESM.pdf]

File name: Supplementary Data 1
Description: The marker genes of 40 cell clusters. Each cell cluster was compared with other clusters to obtain the most up-regulated genes. Statistical testing was performed by a two-sided Wilcoxon test. The P-values were corrected with Benjamini-Hochberg adjustment.

File name: Supplementary Data 2
Description: The number of cells per cell cluster, per patient, or per tissue source.

File name: Supplementary Data 3
Description: The signature genes for each cell cluster used in this study.

File name: Supplementary Data 4
Description: The differential genes of malignant cells between LNMT and PT in 5 represented patients. Statistical testing was performed by a two-sided Wilcoxon test. The P-values were corrected with Benjamini-Hochberg adjustment.

File name: Supplementary Data 5
Description: The GSEA pathway enrichment results for comparing malignant cells between LNMT and PT in 5 represented patients. Statistical testing was performed by permutation test. The P-values were corrected with Benjamini-Hochberg adjustment.

File name: Supplementary Data 6
Description: The shared GSEA pathway across 5 represented patients.
