# Supplementary material for: Single cell profiling of primary and paired metastatic lymph node tumors in breast cancer patients
Source: Nat Commun. 2022 Nov 10;13:6823. doi: 10.1038/s41467-022-34581-2 (PMC9649678; doi:10.1038/s41467-022-34581-2)
Supplement: Supplementary file 10 — Reporting Summary [file 41467_2022_34581_MOESM10_ESM.pdf]

nature portfolio

Corresponding author(s): Zhigao Li, Xiaofan Wei, Hongquan Zhang

Last updated by author(s): Oct 23, 2022

Reporting Summary

Nature Portfolio wishes to improve the reproducibility of the work that we publish. This form provides structure for consistency and transparency in reporting. For further information on Nature Portfolio policies, see our Editorial Policies and the Editorial Policy Checklist.

Statistics

For all statistical analyses, confirm that the following items are present in the figure legend, table legend, main text, or Methods section.

n/a	Confirmed	
☐	☒	The exact sample size (*n*) for each experimental group/condition, given as a discrete number and unit of measurement
☐	☒	A statement on whether measurements were taken from distinct samples or whether the same sample was measured repeatedly
☐	☒	The statistical test(s) used AND whether they are one- or two-sided<br>*Only common tests should be described solely by name; describe more complex techniques in the Methods section.*
☒	☐	A description of all covariates tested
☐	☒	A description of any assumptions or corrections, such as tests of normality and adjustment for multiple comparisons
☐	☒	A full description of the statistical parameters including central tendency (e.g. means) or other basic estimates (e.g. regression coefficient) AND variation (e.g. standard deviation) or associated estimates of uncertainty (e.g. confidence intervals)
☐	☒	For null hypothesis testing, the test statistic (e.g. *F*, *t*, *r*) with confidence intervals, effect sizes, degrees of freedom and *P* value noted<br>*Give P values as exact values whenever suitable.*
☒	☐	For Bayesian analysis, information on the choice of priors and Markov chain Monte Carlo settings
☒	☐	For hierarchical and complex designs, identification of the appropriate level for tests and full reporting of outcomes
☐	☒	Estimates of effect sizes (e.g. Cohen's *d*, Pearson's *r*), indicating how they were calculated

Our web collection on statistics for biologists contains articles on many of the points above.

Software and code

Policy information about availability of computer code

Data collection	Cell Ranger v. 3.1.0 – counts mode, Barcode Identification, Alignment, Quantification<br>Cell Ranger v. 3.1.0 – VDJ mode, Barcode Identification, Alignment, Filter, Quantification<br>space ranger v. 1.2.0 Barcode Identification, Alignment, Filter, Quantification
Data analysis	Source code can be found on online website link: https://github.com/bio-liucheng/brca-singlecell. Packages and version used in this study including:<br>cellphoneDB 2.1.2<br>ComplexHeatmap 2.2.0<br>edgeR 3.26.8<br>GSVA 1.32.0<br>infercnv 1.3.3<br>louvain 0.6.1<br>Monocle 2 2.6.3<br>pheatmap 1.0.12<br>R 3.6.1<br>scanpy 1.6.0 1.4.1<br>scCancer 2.1.0<br>scipy 1.4.1 0.3<br>scrublet 0.2.3

scvelo 0.2.2
Seurat 3.1.5
Seurat 4.0.1
SoupX 1.4.5
survival 3.2-3
velocyto 0.17.17
STARTRAC 0.1.0

For manuscripts utilizing custom algorithms or software that are central to the research but not yet described in published literature, software must be made available to editors and reviewers. We strongly encourage code deposition in a community repository (e.g. GitHub). See the Nature Portfolio guidelines for submitting code & software for further information.

Data

Policy information about availability of data

All manuscripts must include a data availability statement. This statement should provide the following information, where applicable:

- Accession codes, unique identifiers, or web links for publicly available datasets
- A description of any restrictions on data availability
- For clinical datasets or third party data, please ensure that the statement adheres to our policy

The raw data and processed data of single-cell RNA-sequencing data and single-cell TCR sequencing data have been deposited in the Gene Expression Omnibus (GEO) database under accession code GSE167036 [https://www.ncbi.nlm.nih.gov/geo/query/acc.cgi?acc=GSE167036]. The raw data and processed data of spatial transcriptomic data generated in this study have been deposited in the Gene Expression Omnibus (GEO) database under accession code GSE190811 [https://www. ncbi.nlm.nih.gov/geo/query/acc.cgi?acc=GSE190811]. The publicly available single cell dataset used in this study are available from the Gene Expression Omnibus (accession numbers GSE114727 [www.ncbi.nlm.nih.gov/geo/query/acc.cgi?acc=GSE114727]

Human research participants

Policy information about studies involving human research participants and Sex and Gender in Research.

Reporting on sex and gender	All of the patients are female.
Population characteristics	All of the patients are Chinese. We conducted single-cell RNA-seq and single cell TCR-seq experiments with primary tumor and paired lymph node metastasis samples from 8 breast cancer patients by surgical resection. From patient 1 to 8, the ages are 58, 56, 66, 60, 47, 55, 55, 56.
Recruitment	Treatment-naïve female patients with a pathological diagnosis of breast invasive ductal carcinoma associated with lymph node metastasis were recruited. Clinical information was collected after writing informed consents. There are no self-selection bias or any bias that may be present.
Ethics oversight	This study was approved by the Research and Ethical Committee of Harbin Medical University Cancer Hospital and complied with all relevant ethical regulations (IRB:KY2019-08). Written informed consents were obtained from all participants in the study.

Note that full information on the approval of the study protocol must also be provided in the manuscript.

Field-specific reporting

Please select the one below that is the best fit for your research. If you are not sure, read the appropriate sections before making your selection.

☒ Life sciences ☐ Behavioural & social sciences ☐ Ecological, evolutionary & environmental sciences

For a reference copy of the document with all sections, see nature.com/documents/nr-reporting-summary-flat.pdf

Life sciences study design

All studies must disclose on these points even when the disclosure is negative.

Sample size	No sample size calculation was performed, sample sizes were based on accepted conventions and requirement for statistics.
Data exclusions	In TCR analysis, to obtain good quality data, we excluded patient 2 and patient 4 from the downstream analysis because of low T-cell capture rates. Other data were not excluded in downstream analysis.
Replication	All results presented in manuscript were reliably reproduced. Wet lab experiments are representative of multiple independent experiments.
Randomization	The human tissues of breast cancer were collected randomly in primary tumor, while within the lymph node metastasis, we collected tissues with obvious metastasis under the supervision of professional pathologist.
Blinding	Blinding was not relevant with this type of analysis, we collected samples that were available to us. Investigators were blinded to allocation during experiments and outcome assessments.

Reporting for specific materials, systems and methods

We require information from authors about some types of materials, experimental systems and methods used in many studies. Here, indicate whether each material, system or method listed is relevant to your study. If you are not sure if a list item applies to your research, read the appropriate section before selecting a response.

Materials & experimental systems

n/a	Involved in the study
☐	☒ Antibodies
☐	☒ Eukaryotic cell lines
☒	☐ Palaeontology and archaeology
☒	☐ Animals and other organisms
☒	☐ Clinical data
☐	☒ Dual use research of concern

Methods

n/a	Involved in the study
☒	☐ ChIP-seq
☒	☐ Flow cytometry
☒	☐ MRI-based neuroimaging

Antibodies

Antibodies used	Rabbit-CD8A was purchased from Abcam(AB17147): IF,1:200.<br>Rabbit-CD68 were purchased from Abcam(AB213363) :IF,1:200.<br>Rabbit-PLA2G2A were purchased from Invitrogen (PA5-102403) :IF,1:200.
Validation	All antibodies were validated by the manufacturer. https://www.abcam.com/cd8-alpha-antibody-c8144b-ab17147.html; https://www.abcam.com/cd68-antibody-epr20545-ab213363.html; https://www.thermofisher.com/cn/zh/antibody/product/PLA2G2A-Antibody-Polyclonal/PA5-102403

Eukaryotic cell lines

Policy information about cell lines and Sex and Gender in Research

Cell line source(s)	THP-1(1101HUM-PUMC000057)
Authentication	THP-1 was purchased from Cell Resource Center of Peking Union Medical College. No validation technique was used.
Mycoplasma contamination	Cell line has no mycoplasma contamination
Commonly misidentified lines<br>(See ICLAC register)	None of the cell lines used in this study was found in the database of misidentified cell lines

Dual use research of concern

Policy information about dual use research of concern

Hazards

Could the accidental, deliberate or reckless misuse of agents or technologies generated in the work, or the application of information presented in the manuscript, pose a threat to:

No	Yes	
☒	☐	Public health
☒	☐	National security
☒	☐	Crops and/or livestock
☒	☐	Ecosystems
☒	☐	Any other significant area

Experiments of concern

Does the work involve any of these experiments of concern:

No	Yes	
☒	☐	Demonstrate how to render a vaccine ineffective
☒	☐	Confer resistance to therapeutically useful antibiotics or antiviral agents
☒	☐	Enhance the virulence of a pathogen or render a nonpathogen virulent
☒	☐	Increase transmissibility of a pathogen
☒	☐	Alter the host range of a pathogen
☒	☐	Enable evasion of diagnostic/detection modalities
☒	☐	Enable the weaponization of a biological agent or toxin
☒	☐	Any other potentially harmful combination of experiments and agents
